# Sphingomyelinase promotes oxidant production and skeletal muscle contractile dysfunction through activation of NADPH oxidase

**DOI:** 10.3389/fphys.2014.00530

**Published:** 2015-01-21

**Authors:** James A. Loehr, Reem Abo-Zahrah, Rituraj Pal, George G. Rodney

**Affiliations:** Department of Molecular Physiology and Biophysics, Baylor College of MedicineHouston, TX, USA

**Keywords:** ROS, skeletal muscle, Nox2, sphingomyelinase, force

## Abstract

Elevated concentrations of sphingomyelinase (SMase) have been detected in a variety of diseases. SMase has been shown to increase muscle derived oxidants and decrease skeletal muscle force; however, the sub-cellular site of oxidant production has not been elucidated. Using redox sensitive biosensors targeted to the mitochondria and NADPH oxidase (Nox2), we demonstrate that SMase increased Nox2-dependent ROS and had no effect on mitochondrial ROS in isolated FDB fibers. Pharmacological inhibition and genetic knockdown of Nox2 activity prevented SMase induced ROS production and provided protection against decreased force production in the diaphragm. In contrast, genetic overexpression of superoxide dismutase within the mitochondria did not prevent increased ROS production and offered no protection against decreased diaphragm function in response to SMase. Our study shows that SMase induced ROS production occurs in specific sub-cellular regions of skeletal muscle; however, the increased ROS does not completely account for the decrease in muscle function.

## Introduction

A variety of chronic diseases such as chronic heart failure (Doehner et al., 2007; Empinado et al., 2014), inflammatory disease (Wong et al., 2000), and sepsis (Okazaki et al., 2014) have been correlated with muscle weakness. Shingomyelinase (SMase), an enzyme involved in sphingolipid metabolism, has been shown to be elevated during these conditions (Wong et al., 2000; Claus et al., 2005; Empinado et al., 2014). Sphingolipids are abundant in skeletal muscle and their metabolites affect a wide variety of molecular processes (Nikolova-Karakashian and Reid, 2011). SMase induced sphingolipid metabolism results in an increase in ceramide production which has been shown to decrease force production and increase fatigue and muscle atrophy (Ferreira et al., 2010, 2012; De et al., 2012; Empinado et al., 2014).

Reactive oxygen species (ROS) have been shown to play a key role in modulating muscle function (Reid et al., 1993). In contracting skeletal muscle, mitochondria have traditionally been thought to be the major source of ROS; however, more recent evidence indicates that NADPH Oxidase (Nox2) may play a predominant role in ROS generation (Michaelson et al., 2010; Pal et al., 2013, 2014b; Sakellariou et al., 2013, 2014). In non-muscle cells, SMase has been shown to increase both mitochondrial and Nox2-dependent ROS (Sawada et al., 2004; Reinehr et al., 2005); while in skeletal muscle the mitochondria appear to be the primary source (Ferreira et al., 2012).

Until recently, there has been no reliable method to determine the sub-cellular origins of ROS. Traditionally, dichlorofluorescein (DCFH), in combination with various antioxidants, has been used to assess intracellular ROS production and extrapolate oxidant origin. While DCFH is useful for measuring general cytosolic ROS production, it is an irreversible dye that cannot differentiate between subcellular sources of ROS or determine redox state within the cell. Therefore, redox-sensitive green fluorescent probes (roGFP) with an artificial dithiol-disulfide pair inserted into the GFP (Hanson et al., 2004) were developed to function as reversible redox sensors and evaluate the local redox balance within the cell (Dooley et al., 2004; Hanson et al., 2004; Pal et al., 2013). We have shown that ro-GFPs specifically targeted to Nox2 (p47-roGFP) or the mitochondria (mito-roGFP) allow for measurement of site-specific ROS production within skeletal muscle (Michaelson et al., 2010; Pal et al., 2013, 2014a). Therefore, the aim of this project was to determine the specific subcellular site of ROS production in response to SMase stimulation. Our data indicate that Nox2 is a major contributor to ROS production in skeletal muscle (Michaelson et al., 2010; Pal et al., 2013, 2014b; Sakellariou et al., 2013, 2014); therefore, we hypothesized that SMase would induce Nox2-specific ROS and impair muscle function.

## Materials and methods

### *In-vivo* electroporation

C57Bl/6J wild type (WT) and Nox2 knockout (Nox2^−/y^) were purchased from Jackson Laboratories (Bar Harbor, ME) and bred following their breeding strategy. Mitochondrial SOD (MnSOD) transgenic mice were a kind gift from Dr. Pautler and were genotyped by PCR as previously described to verify transgene overexpression (Massaad et al., 2009). Redox sensitive GFP probes were transfected into the flexor digitorum brevis (FDB) of male WT, Nox2^−/y^, and MnSOD mice as previously described (Michaelson et al., 2010) and in accordance with National Institutes of Health guidelines and with the approval of the Institutional Animal Care and Use Committee of Baylor College of Medicine. Briefly, mice between 8 and 12 weeks of age were anesthetized with isoflurane (2%) and hyaluronidase (0.5 mg/ml) dissolved in sterile saline was injected subcutaneously into the foot pad of both hindlimb feet. Two hours later each foot was injected with 20–30 μg of rDNA in PBS; typically the right foot with p47-roGFP and the left foot with mito-roGFP. Two electrodes were placed subcutaneously at the proximal and distal FDB tendons to deliver 20 pulses of 150 V, 20 ms in duration at a frequency of 1 Hz with a square pulse stimulator (S48; Grass Technologies, West Warwick, RI). Mice were returned to their home cage and FDB muscle fibers were isolated 6–8 days later.

### Isolation of FDB fibers

Mice were deeply anesthetized by isofluorane (2%) inhalation and euthanized by rapid cervical dislocation. FDB muscles were surgically isolated and incubated at 37°C in minimal essential media containing 1% Pen Strep (Life Technologies, Grand Island, NY) and 0.4% Collagenase A (Roche Applied Science, Indianapolis, IN) for 2.0 h. FDB muscles were transferred to a serum containing media (10%, Atlanta Biologicals) without collagenase and triturated gently to release single fibers. Single fibers were then incubated in 21% O_2_/5% CO_2_ at 37°C until used, typically 12–36 h later.

### ROS measurements

Prior to experiments, fibers were plated in 96 well dishes (Greiner Bio One, Monroe, NC) on ECM gel from Engelbreth-Holm-Swarm murine sarcoma (Sigma, St. Louis, MO). Intracellular ROS, in unstimulated FDB fibers, was measured using 6-carboxy-2′,7′-dichlorodihydrofluorescein diacetate (DCFH-DA) (Invitrogen, Carlsbad, CA) or electroporated site-specific sub-cellular redox sensitive ROS probes targeted to Nox2 (p47-roGFP) or the mitochondria (mito-roGFP). All FDB fibers were imaged at baseline (no drug) and then incubated for 30 min at 37°C with either vehicle control (0.13% Glycerol final) or SMase (0.5 U/ml). Cells were imaged after 30 min, placed back in the incubator for another 30 min and imaged again at 60 min. To obtain maximal oxidation, p47-roGFP and mito-roGFP electroporated fibers were exposed to 100 μM H_2_O_2_ for 5 min followed by dithiothreitol (DTT, 10 mM) to obtain maximum reduction. Regions of interest (ROI) were drawn on the cell and in the background. Ratio images (403/470) were created by subtracting the mean background ROI from the mean cell ROI.

### DCFH-DA loading and treatment of single FDB fibers

Plated FDB fibers were washed with 4-(2-Hydroxyethyl) piperazine-1-ethanesulfonic acid (HEPES) buffered Ringer's solution containing (in mM): 146 NaCl, 4.7 KCl, 0.6 MgSO4, 1.8 CaCl, 1.6, NaHCO3, 0.13 NaH2PO4, 7.8 glucose, 20.0 HEPES, pH 7.3 and incubated with DCFH-DA (5 μM) for 30 min at 37°C. The fibers were then washed with Ringer's solution and the dye was allowed to de-esterify for 20 min at 37°C prior to fluorescence microscopy. To prevent light induced oxidation of DCFH, all cell-loading and imaging was performed in the dark. In a subset of conditions, prior to DCFH-DA incubation, cells were incubated with a Nox2 peptide inhibitor, gp-91 ds (5 μM; Biosynthesis Inc., Lewisville, TX), for 45 min at 37°C.

### Microscopy

A Sutter Lamda DG-5 Ultra high speed wavelength switcher was used to excite DCF (480 nm) and ro-GFP (403/12 nm and 470/20 nm) fluorescence. DCFH-DA and ro-GFP emission intensity was collected at 510 nm and 535/48 nm, respectively, on a charge coupled device (CCD) Camera (CoolSNAP MYO, Photometrics, Tucson, AZ) attached to an Axio Observer (Zeiss) inverted microscope (40× H_2_O objective, 1.2 NA) at a rate of 0.1 Hz.

### *Ex vivo* force measurements

Diaphragm muscle was surgically dissected from mice and sectioned into diaphragm strips with one end attached to a fixed hook and the other to a force transducer (F30, Harvard Apparatus) using silk suture (4-0) in a physiological saline solution continuously gassed with 95% O_2_–5% CO_2_ at 25°C. Diaphragm strips were incubated at 37°C for 15 min and optimal muscle length (L_*o*_) and voltage (V_max_) were adjusted to elicit maximum twitch force. SMase (0.5 U/ml) or vehicle control (0.13% glycerol final) was administered to the muscle bath and force at 300 Hz was monitored over time at 0, 30, and 60 min with pulse and train durations of 0.5 and 400 ms, respectively. After 60 min of incubation force-frequency characteristics were measured at stimulation frequencies of 1, 5, 10, 20, 40, 60, 80, 120, 150, 200, and 300-Hz every minute with pulse and train durations of 0.5 and 250 ms. Following a 5 min rest period fatigue was assessed using a matched force protocol in which frequency was adjusted so force generated by drug treated tissue matched the force of vehicle control at 40 Hz with pulse and train durations of 0.5 and 400 ms and a train rate of 0.5 Hz. ROS has been suggested to decrease force by altering Ca^2+^ sensitivity; therefore a frequency on the steep part of the force frequency relationship (40 Hz) was chosen to determine if inhibiting SMase induced ROS protected against decreased muscle force. At the end of the contractile protocol muscle length was measured using a hand-held electronic caliper, fiber bundles were removed from the bath and trimmed of excess bone and connective tissue, blotted dry, and weighed. Muscle weight and Lo were used to estimate cross-sectional area and absolute forces expressed as N/cm^2^ (Close, 1972).

### Data analysis

Data are reported as mean ± SEM, unless otherwise specified. A One-Way RM ANOVA was used to measure statistical differences within a group and a Two-Way RM ANOVA was used for between group differences. Tukey's *post-hoc* test was used when statistical differences were identified. Statistical analysis was performed in Origin Pro (OriginLab Corporation, Northhampton, MA) with significance set *a priori* at *p* ≤ 0.05.

## Results

### Effect of SMase stimulation on general ROS production

**Figure 1:**
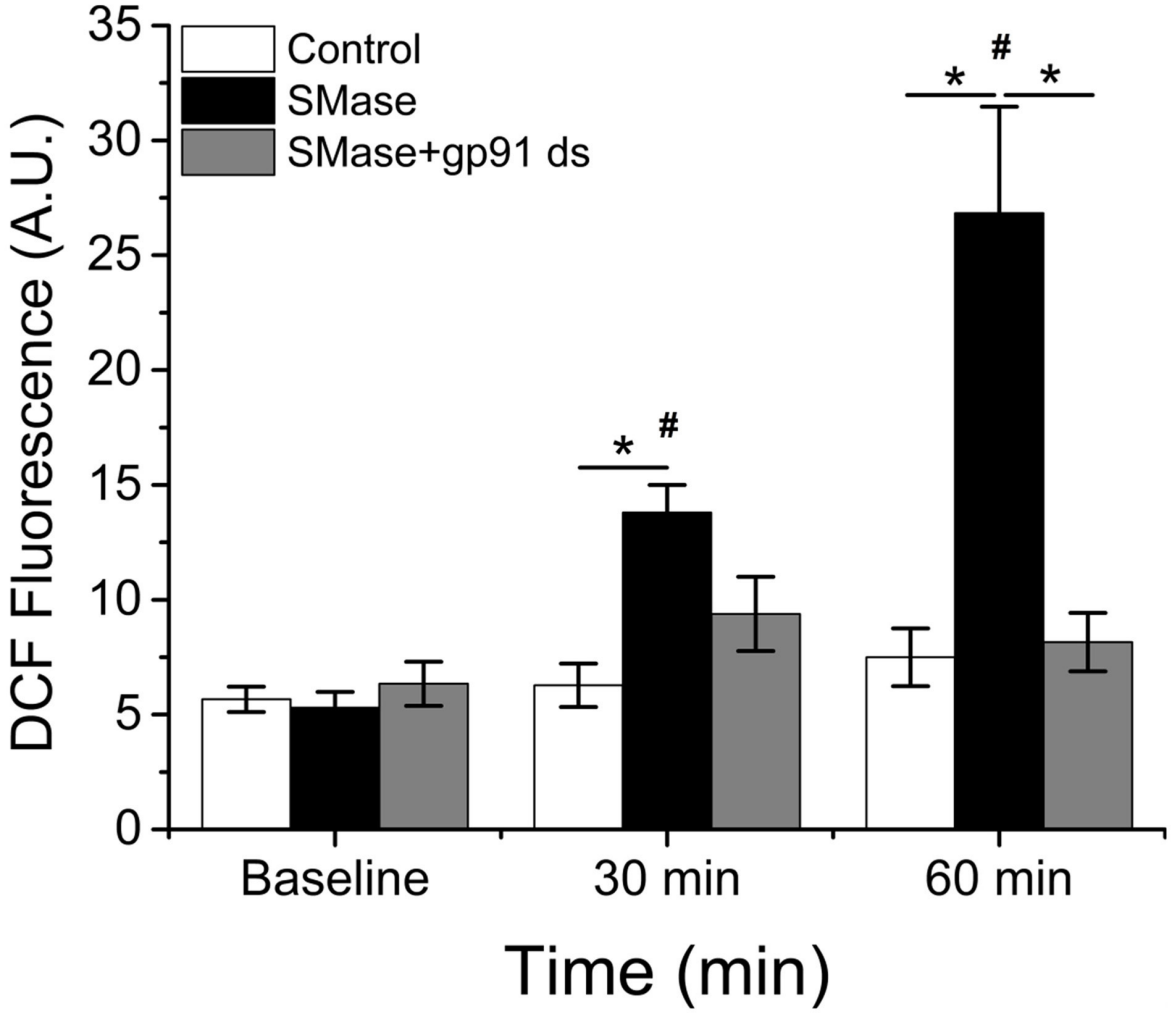
**SMase increases ROS production through Nox2 in FDB fibers**. SMase increased DCF fluorescence in WT FDB fibers (black bars), which could be completely inhibited upon pre-incubation with gp91-ds (gray bars). There was no time-dependent change in the absence of SMase (white bars). *p* ≤ 0.05 ^*^significantly different from vehicle treated control, ^#^significantly different from baseline in at least n_animals_ = 3, n_cells_ = 31.

To assess the role of Nox2 in general non-specific intracellular ROS production, we measured changes in DCF fluorescence in FDB fibers in the presence or absence of the Nox2 specific peptide inhibitor gp91-ds (Rey et al., 2001; Csanyi et al., 2011; Pal et al., 2013). SMase resulted in an increase (*p* ≤ 0.05) in DCF fluorescence at 30 min and a further increase at 60min, which was inhibited by pre-incubation with gp91-ds (Figure 1). There was no change in control fibers over the same time. These data suggest that in skeletal muscle, SMase stimulates Nox2 dependent ROS production.

### Subcellular ROS sensors targeted to NADPH oxidase (Nox2) and the mitochondria

**Figure 2:**
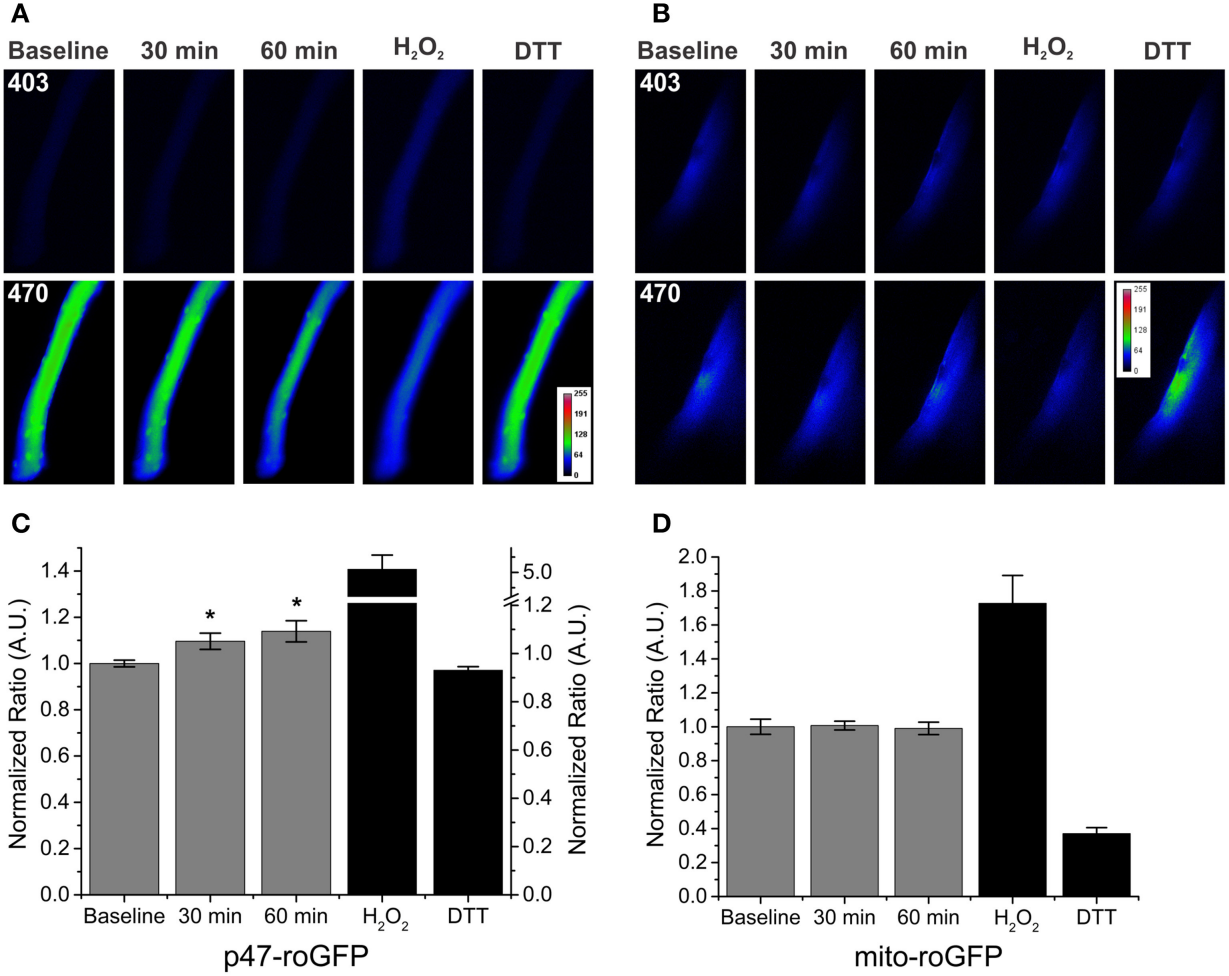
**SMase increased Nox2 ROS production in WT FDB fibers with no effect on mitochondrial ROS. (A,B)** Representative images of SMase induced changes in p47-roGFP and mito-roGFP fluorescence, respectively. **(C)** Nox2 ROS (p47-roGFP) in FDBs from WT animals was elevated following SMase at both 30 and 60 min. H_2_O_2_ and DTT (100 μM and 10 mM, black bars) resulted in further oxidation and reduction of p47-roGFP. **(D)** Mitochondrial ROS (mito-roGFP) did not change in response to SMase. H_2_O_2_ and DTT (100 μM and 10 mM, black bars) resulted in further oxidation and reduction of mito-roGFP. Fluorescence values were normalized to non-drug treated baseline measurements. *p* ≤ 0.05 ^*^significantly different from baseline in at least n_animals_ = 4, n_cells_ = 11.

While our data indicate a role for Nox2 in SMase induced ROS production, others have indicated a potential role for mitochondrial ROS generation (Sawada et al., 2004; Nikolova-Karakashian and Reid, 2011; Ferreira et al., 2012). Therefore, we used redox sensitive GFP probes targeted to Nox2 (p47-roGFP) and the mitochondria (mito-roGFP) to determine the localization of subcellular ROS production. Similar to DCFH, in the absence of SMase, we saw no time dependent effects of ROS production using the p47-roGFP or mito-roGFP ROS sensors (data not shown). SMase exposure increased Nox2 derived ROS following 30 and 60 min (Figure 2C), while it had no effect on increasing mitochondrial oxidant burden (Figure 2D). Representative images demonstrate the change in fluorescence of both p47-roGFP (Figure 2A) and mito-roGFP (Figure 2B) in response to SMase. The use of site-specific redox probes further confirms ROS is produced through Nox2, with no additional mitochondrial contribution.

### Genetic ablation of Nox2 prevents increased ROS production

**Figure 3:**
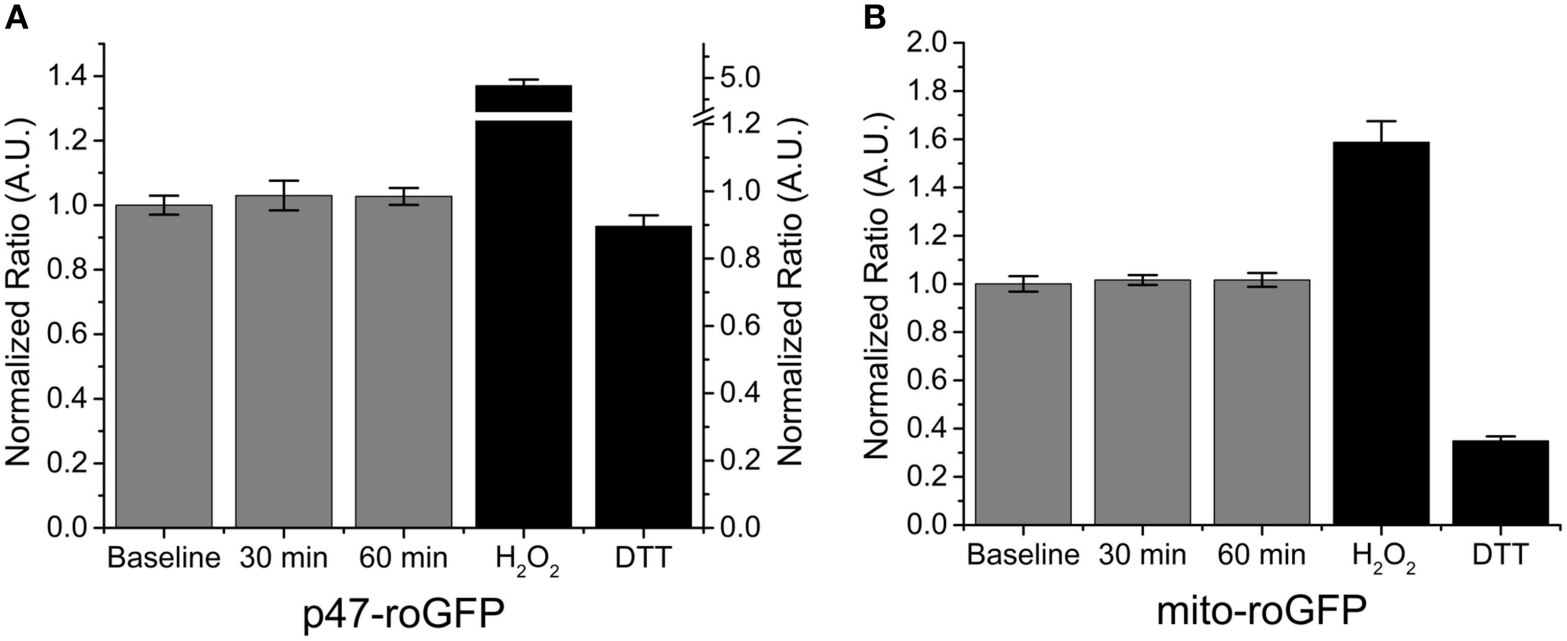
**Genetic deletion of Nox2 prevents SMase induced ROS. (A)** Nox2 ROS (p47-roGFP) was not altered with SMase in FDBs from Nox2^−/y^ mice. **(B)** Mitochondrial ROS (mito-roGFP) did not change in response to SMase. H_2_O_2_ and DTT (100 μM and 10 mM, black bars) resulted in further oxidation and reduction of both p47-roGFP and mito-roGFP. Fluorescence values were normalized to non-drug treated baseline measurements in at least n_animals_ = 4, n_cells_ = 12.

Our data indicate that Nox2 plays a central role in SMase induced ROS production in skeletal muscle. To further confirm the role of Nox2 we assessed the effects of SMase stimulated ROS production in FDB fibers from Nox2 deficient (Nox2^−/y^) mice. SMase induced oxidation of p47-roGFP was completely abolished (Figure 3A) and, consistent with our previous findings, there was no change in the oxidation of mito-roGFP (Figure 3B). The lack of ROS production in Nox2^−/y^ animals further supports the role of Nox2 as the source of ROS production in adult skeletal muscle exposed to SMase.

### MnSOD overexpression does not prevent SMase induced ROS production

**Figure 4:**
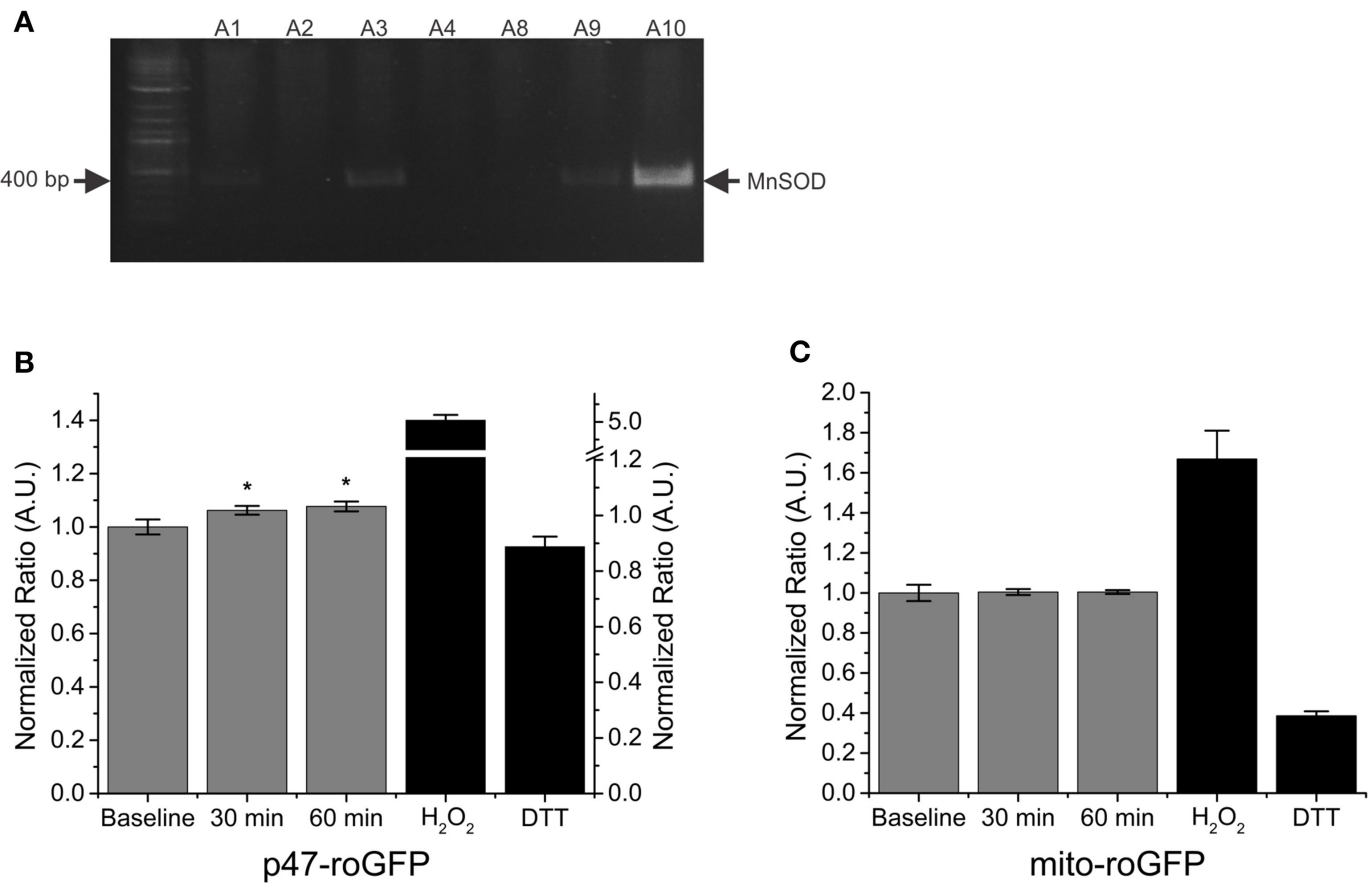
**SMase increased Nox2 ROS production in FDB fibers from MnSOD overexpressing mice. (A)** Representative DNA gel verifying MnSOD transgene overexpression. **(B)** SMase increased Nox2 ROS (p47-roGFP) in muscle from MnSOD overexpressing animals at both 30 and 60 min. **(C)** Mitochondrial ROS (mito-roGFP) did not change in response to SMase. H_2_O_2_ and DTT (100 μM and 10 mM, black bars) resulted in further oxidation and reduction of both p47-roGFP and mito-roGFP. Fluorescence values were normalized to non-drug treated baseline measurements. *p* ≤ 0.05 ^*^significantly different from baseline in at least n_animals_ = 3, n_cells_ = 10.

Previous data have shown increased mitochondrial ROS in response to SMase stimulation (Ferreira et al., 2012). Genetically overexpressing mitochondrial superoxide dismutase (MnSOD) in mice has been shown to reduce contraction induced mitochondrial ROS in skeletal muscle (McClung et al., 2010); therefore, we next wanted to determine if MnSOD overexpression had any effect on ROS production in response to SMase. A representative DNA gel image, verifying MnSOD transgene overexpression, is shown in Figure 4A. In FDBs from MnSOD overexpressing mice, SMase induced an increase in Nox2 (p47-roGFP) ROS production (Figure 4B), while no difference was detected in mitochondrial ROS production (Figure 4C). These results provide further support that SMase increases ROS production through Nox2, while mitochondrial ROS production is not altered.

### Genetic deletion of Nox2 partially protects against SMase induced force decrement

**Figure 5:**
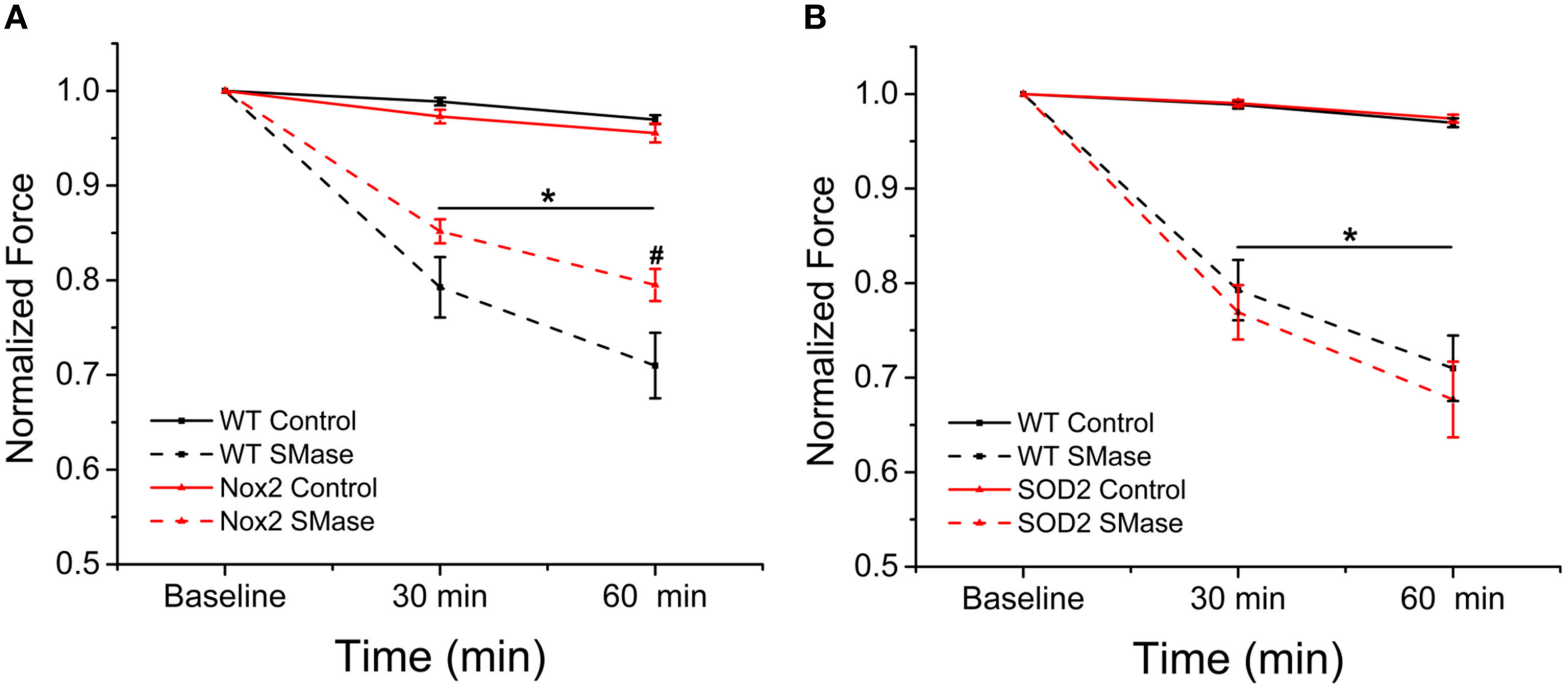
**Genetic deletion of Nox2 protected against diaphragm force decrement over time**. Peak tetanic force was decreased at both 30 and 60 min following SMase administration compared with respective controls. **(A)** Genetic deletion of Nox2 protected against SMase induced force loss compared with WT. Black solid = WT control, Black dash = WT SMase, Red solid = Nox2^−/y^ control, Red dash = Nox2^−/y^ SMase. **(B)** Overexpressing MnSOD did not affect force decrement compared to WT. Black solid = WT control, Black dash = WT SMase, Red solid = MnSOD control, Red dash = MnSOD SMase. Force values at 30 and 60 min were normalized to force measured in the absence of SMase (baseline). *p* ≤ 0.05 ^*^All genotypes significantly different from respective control, ^#^Nox2^−/y^ SMase significantly different from WT SMase in at least n_animals_ = 6.

SMase induced ROS has been shown to decrease force in skeletal muscle (Ferreira et al., 2010, 2012). Our previous data indicate decreased ROS production in Nox2^−/y^ animals in response to SMase; therefore, we wanted to determine whether genetically inhibiting Nox2-dependent ROS production would provide protection against SMase induced force depression in diaphragm strips. In the absence of SMase, there was a non-significant (<4%) time dependent change in force over 60 min (Figures 5A,B). SMase decreased tetanic force (*p* ≤ 0.05) in diaphragm strips from WT, Nox2^−/y^, and MnSOD overexpressing mice compared to their respective controls at 30 and 60 min (Figures 5A,B). Nox2 deficient animals conferred partial protection against the force decrement observed in the diaphragm of WT mice at 60 min (Figure 5A), while the MnSOD overexpressing mice offered no protection in force decrement (Figure 5B). While not statistically significant (*p* ≤ 0.08), there was a trend toward protection against force decrement in the Nox2^−/y^ animals at 30 min.

**Figure 6:**
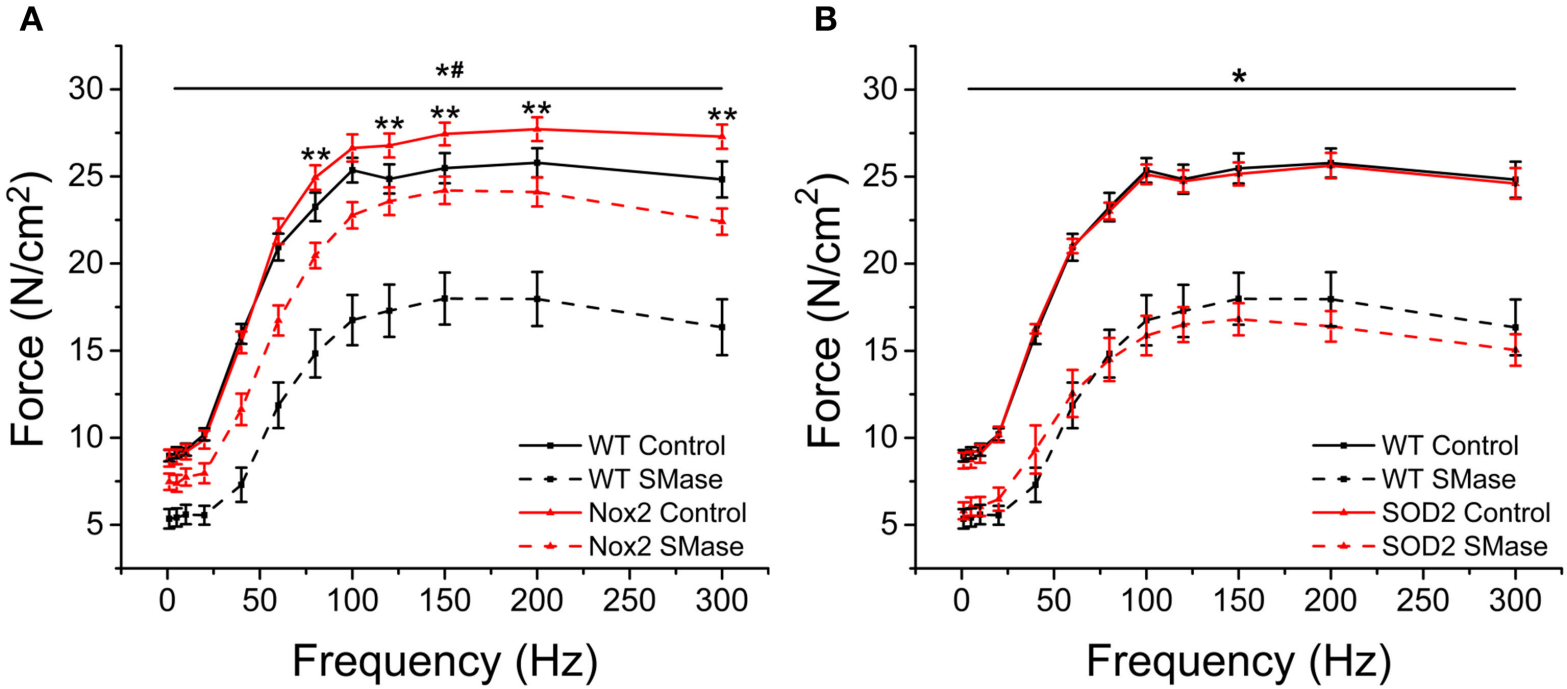
**Nox2 deletion protected against SMase induced decreased force**. Force was decreased at all frequencies following SMase administration compared with respective controls. **(A)** SMase induced force loss was prevented in diaphragm muscle from Nox2^−/y^ mice compared to WT at all frequencies. Nox2^−/y^ controls produced greater force at higher frequencies compared with WT control. Black solid = WT control, Black dash = WT SMase, Red solid = Nox2^−/y^ control, Red dash = Nox2^−/y^ SMase. **(B)** MnSOD overexpression did not prevent force loss at any frequency compared to WT SMase. Black solid = WT control, Black dash = WT SMase, Red solid = MnSOD control, Red dash = MnSOD SMase. *P* ≤ 0.05 ^*^All genotypes significantly different from respective control, ^#^Nox2^−/y^ SMase significantly different from WT SMase, ^**^Nox2 control significantly different from WT control in at least n_animals_ = 4.

SMase induced force decrements were observed across all frequencies of stimulation (Figure 6) Genetic deletion of Nox2 protected against SMase induced force loss at all frequencies of stimulation, with an approximate 51 and 55% protection against twitch and peak tetanic force loss, respectively (Figure 6A). Interestingly, diaphragm from Nox2^−/y^ mice displayed enhanced force production compared to WT animals at higher frequencies of stimulation (Figure 6A), suggesting that Nox2 specific ROS regulates muscle function even under basal conditions. Unlike the protection observed by eliminating Nox2 dependent ROS, no protection was evident in muscle from MnSOD overexpressing mice (Figure 6B), confirming our previous data that SMase does not result in a significant increase in ROS production from the mitochondria. Our data indicate that eliminating SMase stimulated Nox2-dependent ROS confers protection against force decrement and that Nox2-specific ROS may play a role in basal muscle function.

### Nox2 does not confer protection against SMase induced fatigue

**Figure 7:**
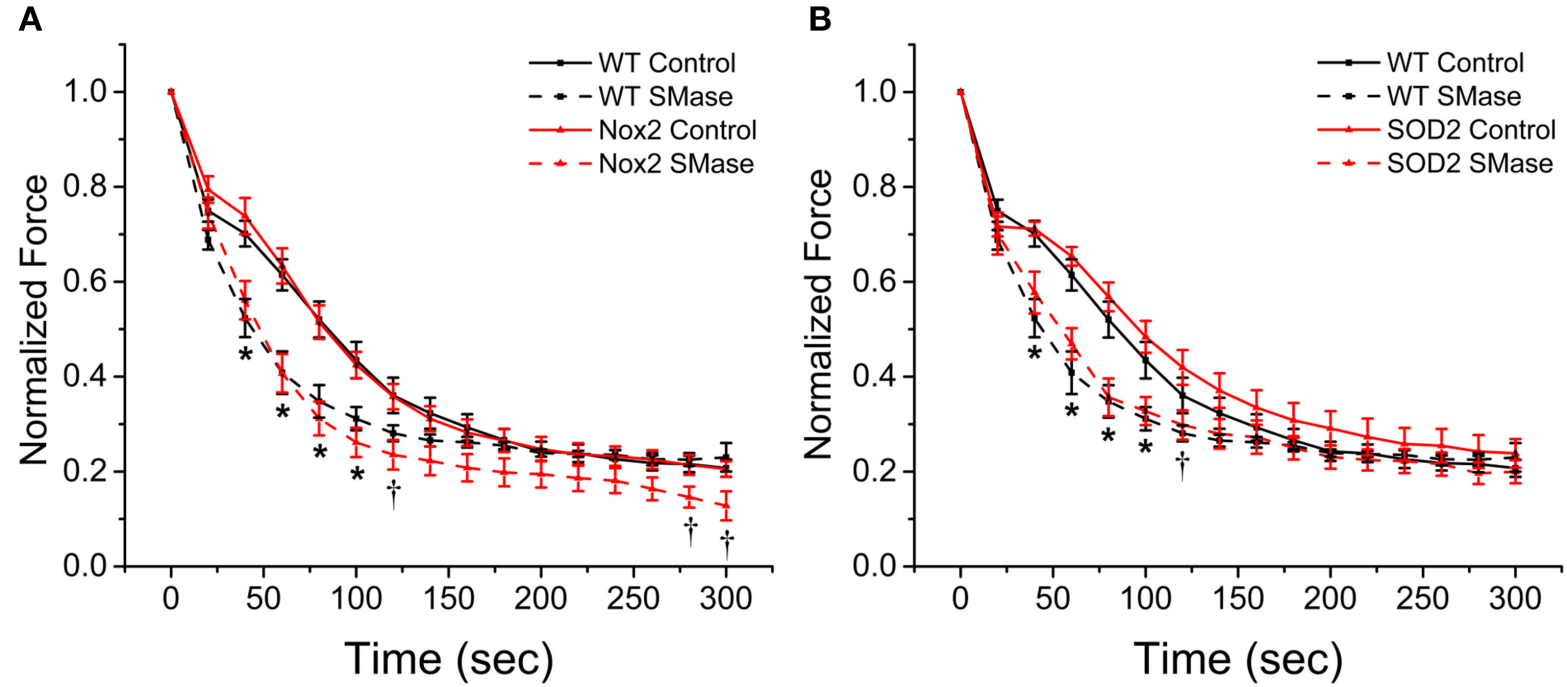
**Genetic ablation of Nox2 or MnSOD overexpression does not protect against SMase induced fatigue**. All genotypes fatigued approximately 75–80%. SMase induced an increased rate of fatigue in diaphragm from WT (**A,B**, black dashed), Nox2^−/y^ (**A**, red dashed), and MnSOD (**B**, red dashed) compared with their respective controls (solid lines). All force values were normalized to initial force. *p* ≤ 0.05 ^*^All genotypes significantly different from respective control, ^†^Nox2^−/y^ and MnSOD significantly different from respective controls in at least n_animals_ = 6.

Oxidants have been shown to promote skeletal muscle fatigue (Moopanar and Allen, 2005; Bruton et al., 2008) and SMase has been shown to increase fatigue in an oxidant dependent manner (Ferreira et al., 2010). Our results show that Nox2 deficiency decreases SMase induced oxidant production and offers partial protection against the decrease in force observed in WT animals; therefore, we wanted to determine if it also would protect against SMase induced fatigue. In the vehicle treated control groups, neither diaphragm from the Nox2^−/y^ nor the MnSOD overexpressing mice showed altered fatigue compared with WT. Following SMase administration, there was an increase in the rate of fatigue for diaphragm from WT, Nox2^−/y^ and MnSOD overexpressing mice (Figures 7A,B). Neither the Nox2 knockout nor the MnSOD overexpressing mice conferred any protection against increased rate of fatigue (Figures 7A,B). The fatigue index, calculated as the last tetanus divided by the first tetanus, indicated diaphragm strips from all animals fatigued approximately 75–80%. These data suggest that fatigue observed in the presence of SMase is likely independent of ROS.

## Discussion

SMase, an enzyme which catalyzes the production of metabolites that affect a number of cellular functions (Nikolova-Karakashian and Reid, 2011), has been shown to be elevated in response to a variety of diseases (Wong et al., 2000; Claus et al., 2005; Empinado et al., 2014). Increased SMase results in increased ROS production, which has been thought to negatively impact muscle force and promote fatigue (Ferreira et al., 2010, 2012). Previous research has shown that SMase can induce either mitochondrial or Nox2 ROS in non-muscle cells (Sawada et al., 2004; Reinehr et al., 2005) while recent indirect evidence suggests SMase induced ROS in skeletal muscle was generated by the mitochondria (Ferreira et al., 2012). The majority of ROS production in skeletal muscle has generally been assumed to be mitochondrial; however, more recent evidence by our lab and others implicate Nox2 as a major source of ROS in skeletal muscle (Michaelson et al., 2010; Pal et al., 2013, 2014b; Sakellariou et al., 2013, 2014). Using site-specific subcellular redox sensors and various genetic animal models, we have shown that SMase induces ROS through Nox2 and eliminating Nox2-dependent ROS partially recovers muscle function.

Using the general ROS probe, DCFH, and a Nox2-specific peptide inhibitor (gp91-ds), we show that Nox2 plays a significant role in SMase induced ROS production. However, since DCFH is a non-specific, irreversible probe that does not allow for the evaluation of cellular redox homeostasis, there is still uncertainty as to the source of ROS within the cell and whether there is a true shift in the redox balance upon SMase exposure. The recent development of reversible, site-specific subcellular redox probes allows for a more thorough evaluation of the source of ROS production and redox balance in response to various stimuli or in response to different disease states. Using ROS sensors specifically targeted to Nox2 (p47-roGFP) and the mitochondria (mito-roGFP), we were able to show that SMase increased Nox2 ROS production, but had no effect on mitochondrial ROS in mouse FDB muscle. The lack of response of mito-roGFP to SMase cannot be attributed to the failure of the probe to respond, as the fluorescence ratio increased approximately two-fold upon oxidation (100 μM H_2_O_2_). These findings are in disagreement with other reports, which suggest SMase induces mitochondrial ROS production in the mouse diaphragm. Using an antioxidant peptide targeted to the inner mitochondrial membrane (SS-31) Reid and colleagues have suggested that SMase induces ROS generation from the mitochondria in mouse diaphragm muscle, which then diffuses into the cytosol where it is detected by the non-specific ROS probe DCFH (Ferreira et al., 2012). While SS-31 has been shown to localize to the mitochondria Szeto and colleagues have also shown that it also localizes to the cytosol (Zhao et al., 2004). This would allow SS-31 to act as a cytosolic ROS scavenger; reducing SMase induced ROS production generated by Nox2. Using pharmacological inhibition of Nox2 (gp91-ds) and genetic deletion of Nox2 (Nox2^−/y^), our results confirm that SMase induces ROS production by activating Nox2 in skeletal muscle. Furthermore, MnSOD overexpressing mice did not confer protection against SMase induced oxidation of our Nox2 redox biosensor.

Another potential confounding variable is that mitochondrial content varies in different muscles based on fiber type composition. Ferreira et al. (2012) used mouse diaphragm, which is comprised of 42–49% oxidative (Type I and IIA) fibers with the mitochondria encompassing approximately 34% of the fiber volume (Gamboa and Andrade, 2010; Guido et al., 2010; Pal et al., 2014b). We evaluated ROS production in FDB fibers, which have been reported to contain approximately 30% oxidative fibers (Gonzalez et al., 2003) with a mitochondrial volume between 10 and 36% of the fiber volume (Bruton et al., 2003; Laker et al., 2014). However, if SMase was able to induce a shift in the mitochondrial redox balance in muscle with a higher mitochondrial content (i.e., diaphgram), one would predict that overexpressing MnSOD would protect against SMase induced dysfunction, which was not observed in our studies. Therefore, we believe that differences in fiber type or mitochondrial content do not affect SMase induced mitochondrial ROS production.

ROS has been shown to be a potent modifier of skeletal muscle contractility and fatigue by modulating myofilament calcium sensitivity (Reid et al., 1993; Andrade et al., 1998; Callahan et al., 2001; Moopanar and Allen, 2005; Bruton et al., 2008). In the present study, eliminating SMase induced ROS provided partial protection against decreased force and had no effect on fatigue. It is unlikely that reactive nitrogen species, another source of oxidants in skeletal muscle, could account for the continued force decrement, as SMase had no effect on NO activity and blocking NO activity had no effect on SMase induced force decrement (Ferreira et al., 2010). Sarcoplasmic reticulum (SR) calcium release has been shown to play an important role in skeletal muscle function (Ortenblad et al., 2000; Allen et al., 2008). The direct metabolite of SMase activity, ceramide, can be further metabolized by ceramidase into sphingosine. Sphingosine has been shown to decrease SR calcium release (Betto et al., 1992; Sabbadini et al., 1992; McDonough et al., 1994) by decreasing ryanodine receptor open probability (Needleman et al., 1997; Sabbadini et al., 1999). Increased levels of sphingosines have also been shown to decrease myocyte calcium transients, cell shortening, and contractile function, (Webster et al., 1994; Oral et al., 1997; Friedrichs et al., 2002; Favory et al., 2004) in addition to promoting muscle fatigue (Danieli-Betto et al., 2000). Therefore, SMase may affect muscle function through a non-ROS dependent mechanism. We are currently investigating the role of these potential mechanisms in skeletal muscle force depression and fatigue.

In summary, we have shown that SMase increases Nox2 dependent ROS and has no effect on mitochondrial ROS production. Genetically deleting or pharmacologically inhibiting Nox2 reduced SMase stimulated ROS production to control levels. In addition, Nox2 inhibition provided protection against decreased force production induced by SMase; however, there was no effect on muscle fatigue. This indicates that SMase, through its downstream metabolites, may affect contractile function in a ROS independent manner, potentially through modifying SR calcium release.

### Conflict of interest statement

The authors declare that the research was conducted in the absence of any commercial or financial relationships that could be construed as a potential conflict of interest.
